# The Role of Liquid Biopsy in the Diagnosis and Prognosis of WHO Grade 4 Astrocytoma

**DOI:** 10.7759/cureus.41221

**Published:** 2023-06-30

**Authors:** Taher Halawa, Saleh Baeesa, Motaz M Fadul, Adnan A Badahdah, Maryam Enani, Amany A Fathaddin, Dania Kawass, Alaa Alkhotani, Basem Bahakeem, Maher Kurdi

**Affiliations:** 1 Department of Pediatrics, Faculty of Medicine King Abdulaziz University, Rabigh, SAU; 2 Department of Neuroscience, King Faisal Specialist Hospital and Research Centre, Jeddah, SAU; 3 Department of Pathology, Faculty of Medicine King Abdulaziz University, Rabigh, SAU; 4 Department of Internal Medicine, University of Jeddah, Jeddah, SAU; 5 Department of Surgery, King Abdulaziz University Hospital, Jeddah, SAU; 6 Department of Pathology, College of Medicine, King Saud University, Riyadh, SAU; 7 Department of Pathology, King Saud University Medical City, Riyadh, SAU; 8 Department of Family Medicine, Faculty of Medicine King Abdulaziz University, Jeddah, SAU; 9 Department of Pathology, Umm Al-Qura University, Makkah, SAU; 10 Department of Internal Medicine, Umm Al-Qura University, Makkah, SAU

**Keywords:** astrocytoma, mirna, crna, cdna, ctc, liquid biome

## Abstract

Liquid biopsy, as a non-invasive diagnostic tool, has recently gained significant attention in the field of oncology. It involves the analysis of various biomarkers present in bodily fluids, such as blood or cerebrospinal fluid, to provide information about the underlying cancer. In the case of WHO grade 4 astrocytomas, liquid biopsy has the potential to significantly impact the diagnosis and prognosis of this aggressive malignant brain tumor. By detecting specific genetic mutations, such as IDH1 or EGFR, and monitoring levels of circulating tumor DNA, liquid biopsy can aid in the early detection and monitoring of disease progression. This innovative approach is gradually being acknowledged as a less invasive and cost-effective procedure for cancer diagnosis and management to improve patient outcomes and quality of life. Various kinds of biomarkers circulating in cerebrospinal fluid (CSF), such as circulating tumor cells (CTC) and different types of nucleic acids like cell-free DNA (cfDNA), cell-free RNA (ctRNA), and microRNAs (miRNA), have been identified. These biomarkers, which require dependable detection methods, are comparatively simple to obtain and allow for repeated measurements, making them significantly superior for disease monitoring. This review aims to compare the latest liquid biopsy analysis tools for both CSF and plasma in the central nervous system.

## Introduction and background

**Figure 1:**
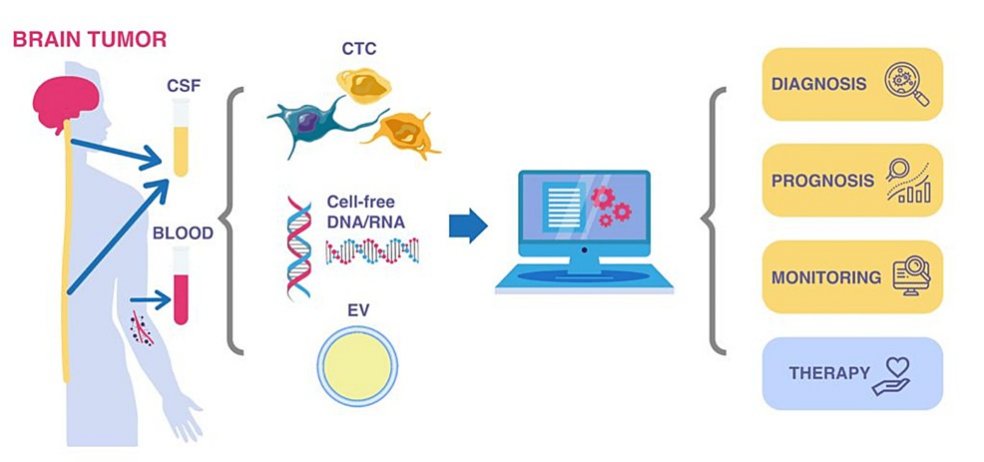
Diagram describing how the circulating tumor cells (CTC) obtained from CSF or blood assists in the diagnosis and monitoring of disease progression EV: extracellular vesicles Image credit: authors

Liquid biopsy is a general scientific term that refers to tests done on any biological fluid (biofluid) obtained from the body including blood, urine, and cerebrospinal fluid (CSF). This sample possibly contains a few tumor cells or tumor-related biomolecules like circulating tumor cells (CTCs), cell-free DNA (cfDNA), circulating tumor DNA (ctDNA), cell-free RNA (ctRNA), microRNAs (miRNAs), exosomes, tumor-educated platelets (TEPs), fragmented peptides, or whole proteins (Figure 1).

The concept of using liquid components to identify circulating tumor molecules is not novel. It dates back to as early as 1869 when CTCs were first discovered in the blood of a deceased cancer patient [1]. The ctDNA was also discovered in the plasma of lung cancer patients [2]. Even though there is a scientific comprehension of tumor biomarkers in bloodstream circulation, the necessary tools to fully utilize the potential of liquid biopsy in cancer management were not accessible until recently.

In the last 10 years, research on liquid biopsy, especially non-invasive detection and monitoring of cancer using biomarkers, has risen dramatically. This is due to the development of highly sensitive analytical techniques for measuring and identifying these biomolecules in biofluids [3]. For the diagnosis of solid cancers, collecting biofluid non-invasively is beneficial for most patients. However, for those with central nervous system (CNS) tumors, the current diagnostic practices may not be as advantageous. In cases where a new CNS tumor is detected by neuroimaging, the standard approach is to obtain an intraoperative tissue diagnosis, with or without maximal safe tumor resection, for cytoreduction [4]. There is a significant danger associated with this procedure that could result in severe neurological deficits and significantly impact the quality of life for patients. It's worth noting that many patients have tumors located in critical areas of the brain, such as regions responsible for speech and motor function, which could increase the risk of neurological deficits [5].

The invasive procedure is generally necessary because several CNS tumor types have similar appearances on neuroimaging. Clinical and radiological examinations alone are insufficient to identify the tumor's histogenesis and molecular components [6]. While liquid biopsy has demonstrated significant clinical applications for various types of cancer, such as breast and colorectal cancers, that have contributed to improving cancer treatment, there has been limited progress in validating circulating tumor biomarkers in aggressive brain tumors, particularly WHO grade 4 astrocytoma [7].

WHO grade 4 astrocytoma is the most common primary malignant brain tumor in adults [8]. The WHO has recently released the fifth edition of its classification for CNS tumors. This new edition includes a classification system that is based on the isocitrate dehydrogenase 1/2 (IDH1/2) mutation. Consequently, IDH-mutant WHO grade 4 astrocytoma has taken the place of IDH-mutant glioblastoma. Glioblastoma is now referred to as IDH-wildtype glioblastoma [8,9]. The new classification system will enhance comprehension of the biological behavior of malignant glial tumors and how they respond to multimodal treatments. The present standard of care for WHO grade 4 astrocytoma includes surgical resection, followed by radiotherapy. Adjuvant chemotherapy is also administered to minimize tumor recurrence. However, the disease's prognosis is still poor, with five-yer overall survival (OS) rate of 15 months [9]. At present, the essential diagnostic methods used for the diagnosis of WHO grade 4 astrocytoma are neuroimaging and pathological examination of the resected tissue [6]. Molecular testing of gliomas is currently utilized to aid in the diagnosis and classification of the tumor, as well as its cellular histogenesis. For WHO grade 4 astrocytoma, one of the crucial molecular tests to conduct is IDH testing, which is usually performed through immunohistochemistry. However, Sanger sequencing is necessary to accurately identify the exact point mutation in the exome. IDH with other targeted gene mutations explored recently in glioblastomas such as telomerase reverse transcriptase (TERT) mutation, epidermal growth factor (EGF) amplification, or chromosomal 10 or 7 gain/loss can be tested using next-generation sequencing (NGS) from the resected tissue. These techniques are performed after surgical resection of the tumors and are not cost-effective. The possibility of having fragmented DNA or RNA is also high because of the nature of tissue processing. The need for less intervention techniques had become crucial.

In recent years, tumor profiling is been considered a less invasive and cost-effective procedure. Different types of circulating biomarkers have been identified in CSF including CTC and cDNA, cRNA, and miRNA [10]. Biomarkers that can be detected in the blood or CSF are easily obtainable, can be measured multiple times, and are a much more effective way to monitor the progression of a disease [6].

## Review

Liquid biopsy versus tissue biopsy: application and features

The primary CNS tumor is usually removed by either neurosurgical resection of the whole or most of the tumor or through stereotactic biopsy. However, these procedures have several limitations, including invasive acquisition, sample preservation, and tumor heterogeneity [11]. Patients with malignant brain tumors are at significant risk of postoperative complications such as in-site hemorrhage and brain swelling, which can damage healthy brain tissue and threaten the patient's life. Additionally, performing repeated tissue biopsies for follow-up can be difficult. Therefore, a less risky alternative or supplement is desirable, as long as the method is highly specific and has an acceptable level of sensitivity. Using a less invasive procedure instead of invasive tissue biopsies to prevent brain tumor regrowth through blood or CSF would considerably decrease the individual risk for the patient, particularly for those with severe comorbidities [12]. Indeed, liquid biopsy is recently considered as an alternative procedure for tissue biopsy; however, its application in clinical practice is still in the nascent stages.

Liquid biopsy is a less invasive process that utilizes general body fluids for clinical diagnosis and follow-up of cancer patients. Unlike traditional biopsies, these body fluids are collected at distant locations from the brain tumor, such as venous blood, CSF via lumbar or cisternal puncture, or samples taken from urine, saliva, bronchial fluid, and vitreous fluid. However, the application of liquid biopsy on CNS tumors is currently challenging because of the strict inflow and outflow through the blood-brain barriers. The therapeutic approach to patient management is the key factor that distinguishes invasive brain biopsy and liquid biopsy. Each method has its own advantages and disadvantages. Invasive tissue biopsies may miss a significant portion of the tumor, which liquid biopsy can detect. On the other hand, a liquid biopsy may not present the entire primary tumor but may represent a more mobile and aggressive part, which can offer an advantage over traditional tissue biopsy [12]. The identification of biomolecules, such as cfDNAs or CTCs, through liquid biopsy, can supplement the standard risk-stratification methods, monitor treatment response, and track disease progression, in contrast to invasive tissue biopsy [13].

Various tumor particles including necrosis, apoptosis, or biomolecules such as cfDNA are circulating through CSF liquid or blood [14]. These materials have undergone extensive research in various body cancers for screening, determining staging, measuring drug resistance, and predicting prognosis. However, as of yet, no clinical application has been introduced for utilizing circulating biomarkers in the management of patients with malignant gliomas. The primary obstacle to using liquid biopsy in high-grade gliomas is the absence of standardized technology, resulting in imprecise findings and insufficient validation. The recent development of more sensitive techniques, such as improved targeted deep NGS, droplet-digital polymerase chain reaction (PCR), and DNA-methylation profiling have improved the utility and applicability of liquid biopsy in clinical practice [15].

CTS: definition, detection, and isolation

CTCs are tumor cells originating from primary tumors, migrating through human blood circulation, and can be identified in systemic blood vessels [16,17]. It was first discovered by Ashworth et al. in 1869 [18]. Ashworth et They found a large number of cancer cells in the blood of a metastatic breast cancer patient while doing the post-mortem examination. CTCs display several advanced characteristics, including epithelial-mesenchymal transition (EMT), which is crucial for supporting their survival in the bloodstream [19]. A molecular typing analysis of CTCs can aid clinicians in diagnosing the recurrence of tumors or metastasis, as well as monitoring the degree of disease progression and response to treatment. Studies have found that only a small percentage of these CTCs, 2.5%, are capable of forming micrometastases. Additionally, only 0.01% of these cells are capable of inducing macrometastases in melanoma [20]. The successful identification of CTCs in the bloodstream invariably hinges on their separation from other circulating cells. Numerous methods have been utilized to isolate CTCs from other cell types, specifically red and white blood cells. The technique for separating CTCs is based on their physical characteristics such as size, deformability, density, and dielectric properties [21]. It is noteworthy that CTCs were found in 20-40% of patients with glioblastoma, a type of brain tumor that does not typically metastasize through the bloodstream [22].

**Figure 2:**
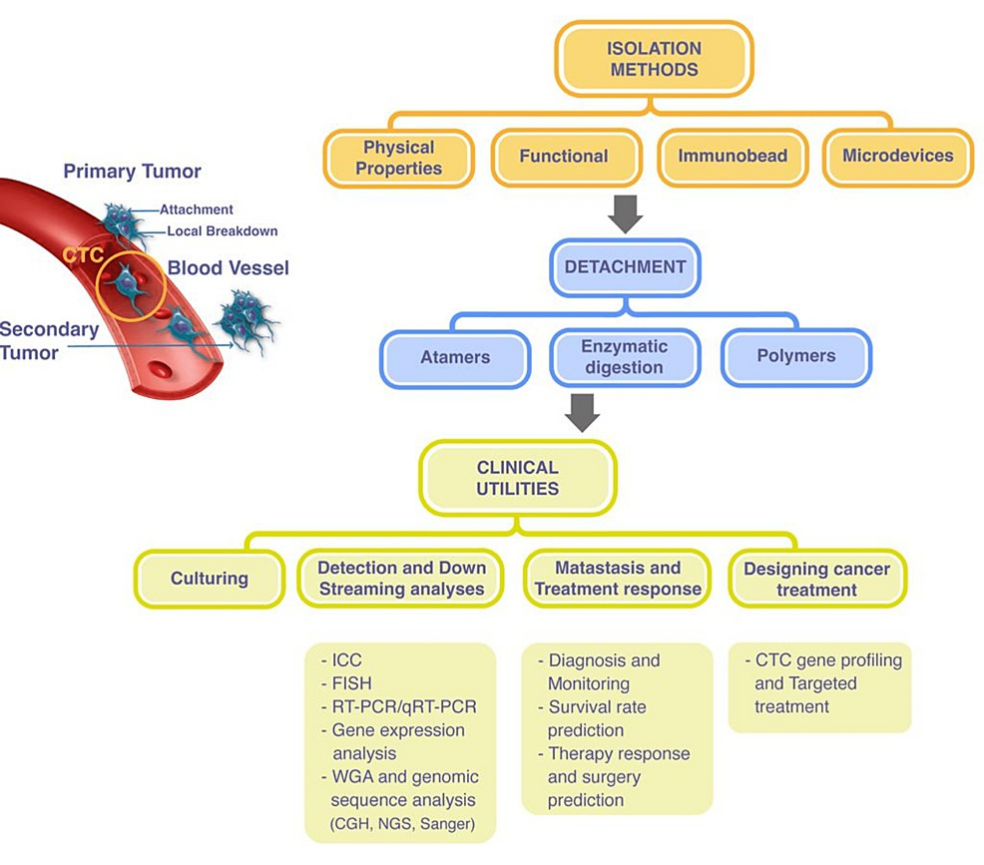
Methods of isolating CTCs from the blood or CSF using different processes. CTCs: circulating tumor cells; ICC: Immunocytochemistry; FISH: fluorescence in situ hybridisation; RT-PCR: reverse transcription-polymerase chain reaction; WGA: whole genome amplification; CGH: comparative genomic hybridization; NGS: next-generation sequencing Image credit: authors

Identifying CTCs is dependent on physical cell properties, such as size, density, and immune and cell-surface electrical properties. As CTCs are a minimal fraction of the total cells in circulating blood, with 1-10 CTCs per mL of whole blood compared to a few million white blood cells and a billion red blood cells, the primary challenge for researchers is purifying CTCs to enable molecular characterization. There are various techniques available for isolating CTCs in the peripheral blood, which can be primarily classified into two categories: biological methods and physical methods. Additionally, there exist hybrid methods that utilize a combination of both these strategies (Figure 2) [23].

The Food and Drug Administration (FDA) had approved a method for CTC enumeration used to predict the outcomes of cancer patient. The CELLSEARCH® Circulating Tumor Cell Kit (Menarini Silicon Biosystems, Inc., Bologna, Emilia-Romagna, Italy) was approved for clinical use in breast, colorectal, and prostatic cancer patients in 2010. It was then produced by Veridex, a Johnson & Johnson (Raritan, New Jersey, United States) company that has now closed [24-26].

Because very few CTCs circulate in CSF fluid, about 1-5 cells/μl, their identification is easy [27]. Identifying CTCs in CSF fluid is easily distinguishable from other cell types, such as immune-derived cells (lymphocytes and monocytes), and other antibodies that penetrate the blood-CSF barrier at the choroid plexus. Nevertheless, these immune cells, primarily central memory CD4+ T cells, serve as immunosurveillance [28]. Several techniques are available to identify CTCs in the bloodstream, which rely on the cell surface type. Epithelial cell adhesion molecule (EpCAM) is extensively expressed on the surface of CTCs derived from cancer cells and has not been observed in normal blood cells [29]. To detect or enrich CTCs, anti-EpCAM antibodies can be used directly while anti-CD45 antibodies can be used to sort out other cells (specific for leukocytes). Various types of nanomaterials, including magnetic nanoparticles (MNPs) and gold nanoparticles (AuNPs), can promote cell adhesion and have been reported for detecting CTCs [30].

CTC isolation technology and quality assurance

Various methods have been employed to detect, enrich, and quantify CTCs, targeting unique physical (size, density, etc.) and biological (tumor markers) attributes in the blood of cancer patients. Utilizing untreated whole blood samples is, in fact, preferable for CTC detection processing (Figure 2). Studies reveal that centrifugation, lysis, or other pre-treatment approaches substantially decrease the count of detectable CTCs in blood samples. As every mL of blood carries over 10^9^ red blood cells, compared to a relatively small number of CTCs, it is vital to isolate CTCs without any interference [31]. To compare different technical methods, the following parameters should be considered during CTCs detection: capture efficiency, enrichment, purity, throughput, cell viability, and release efficiency [32]. Capture efficiency refers to how well a device can collect CTCs from a sample, while enrichment is similar but specifically describes the increase in tumor cell numbers within the sample volume. Purity describes the device's ability to selectively capture CTCs while filtering out interfering cells in the sample. Throughput indicates the volume or count of cells that a device can process from a sample within a given time frame. Cell viability indicates the number of CTCs that remain alive after capture. Release efficiency describes the number of cells that are recovered [33].

Challenges in CTCs utilization

The lack of a standardized and effective method for enriching sufficient CTCs from biological fluids for further analysis is a significant obstacle in CTC detection. Additionally, the technical approaches utilized in evaluating the molecular attributes of CTCs are still unclear, and clinical practice standards have not yet been established or unified. Even with the existence of diverse CTC detection methods, challenges such as processing complexity, lack of specificity, insufficient CTCs in circulation, and difficulty in measuring quality parameters make CTC detection a daunting task. The low CTC frequency in blood has significantly impacted the clinical efficacy of various biomarkers in screening for colorectal cancers, especially in the early stages [34]. For malignant astrocytoma, the challenges are more profound as most CTCs detection strategies rely on antibody-mediated capture targeting cell-surface expression of EpCAM, which is not present in glioma cells [35].

CTCs undergo a process called EMT, initially identified in embryogenesis, wherein epithelial cells are reprogrammed to acquire a mesenchymal phenotype. EMT is crucial in physiological processes like wound healing and development, but it also plays a part in malignant progression [36]. When the reprogramming process begins, CTCs undergo physical transformations that allow them to disseminate over long distances, invade and survive in the bloodstream. Malignant glioma cells that have undergone EMT do not express EpCAM, which makes them undetectable using the Cell-Search approach. Therefore, less specific microfluidic techniques that rely on physical properties are used to isolate CTCs from non-epithelial tumors [6]. Using EpCAM to capture and define CTCs is difficult due to the downregulation of EMT. Confirmatory markers such as cytokeratin “pan-CK” and MUC-1 could be used for targeting CTCs before undergoing physical changes that enable them for long-distance dissemination, invasion, and survival in the bloodstream [37,38].

Innovative tools to improve CTC utilization in WHO grade 4 astrocytoma

Fluorescent In-Site Hybridization (FISH)

Chromosome abnormalities are distinctive features of tumors, and aneuploidy can trigger tumor development. The degree of chromosomal aberrations typically rises with tumor advancement and is linked to an unfavorable prognosis [39]. Tumors that exhibit aggressive clinical behavior are highly susceptible to aneuploidy. Several studies have demonstrated the presence of aneuploidy in chromosome 8 across various solid tumors, such as lung cancer, pancreatic cancer, gastric cancer, colon cancer, and transitional cell carcinoma of the urinary bladder. These results offer a promising opportunity for the identification of CTCs based on aneuploidy [40]. FISH is an effective technique for identifying numerical and structural chromosome aberrations in interphase nuclei of different tumors, using DNA probes specific to each chromosome. In a study by Gao et al., CEP8-FISH was utilized to detect CTCs in the peripheral blood of glioma patients [41]. The identification of CTCs in various pathological subtypes of glioma suggests that CTCs are a shared characteristic of gliomas. Gao et al. discovered that the method employed in detecting glioma CTCs did not depend on surface antigen expression or cell size of glioma cells. Despite this, the findings demonstrated favorable sensitivity [41].

Enrichment Strategy

Due to the rarity of CTCs in circulation, their identification and interpretation are inspiring dilemmas. To improve CTCs detection in body fluids mainly the bloodstream, the process is preceded by an enrichment technique to unmask the low-frequency cells from the myriad of blood cells: white blood cells (5-10 × 106/mL), red blood cells (5-9 × 109/mL) and platelets (2.5-4 × 108/mL). The enrichment technique employed in detecting CTCs capitalizes on their distinct physical and biological characteristics, including size, density, and surface markers. Immunocapturing is the most commonly used approach among various enrichment methods, utilized for both collecting CTCs (positive enrichment) and eliminating blood cell subpopulations (negative enrichment) [42]. Using immunological identification to detect specific tumor markers expressed on the surface of CTCs, positive enrichment is better suited for collecting CTCs from CSF.

cfDNA

Liquid biopsies have evolved from early-stage cancer detection to monitoring tumor progression, evaluating tumor heterogeneity and residual disease, and potentially monitoring the response to various surgical and chemotherapeutic interventions. When cells release DNA into body fluids, it is referred to as cfDNA. If the cells shedding the DNA are cancerous, and undergo apoptosis or necrosis, the DNA is identified as ctDNA. NGS technique can be utilized to detect genomic alterations from these samples [42].

Mandel et al. were the first to publish a description of fragmented cfDNA in human blood [43]. It has been established those specific molecules such as cfDNA, cfRNA, proteins, and metabolites can be found in various biological fluids, such as blood, CSF, urine, saliva, and semen [23,44,45]. cfDNA is composed of around 160 base pair fragments of genetic material. When originating from a tumor, it is referred to as ctDNA and could be identified in the blood and CSF of cancer patients. These ctDNA fragments carry genetic information, demonstrating the tumor's source, along with DNA alterations. Nevertheless, ctDNA has a high turnover rate due to its fast clearance., which makes its detection difficult in cases of low tumor burden [46].

ctDNA

The identification of ctDNA in CSF is regarded as a promising avenue for clinical diagnosis and prognosis in patients with WHO-grade 4 astrocytoma [47]. The levels and composition of cfDNA in the blood are subject to variability based on multiple factors, such as autoimmune diseases, chronic smoking, pregnancy, intense physical activity, and tissue-damaging therapies. Conversely, ctDNA is a more specific marker for tumors. Despite the potential of ctDNA analysis for early cancer diagnosis, existing techniques encounter obstacles in achieving adequate sensitivity for detection [48]. Comparing CTCs with ctDNA's ability to assess methylation will be difficult as FISH and full chromosomal analysis can be done for CTCs. The identification of CTCs and ctDNA in liquid biopsies from cancer patients has the possibility of providing a higher degree of cancer-specificity. However, both CTCs and ctDNA are present in low quantities in circulating biofluids, rendering them unsuitable as clinically relevant diagnostic biomarkers. Generally, ctDNA constitutes less than 1% of the total cfDNA in biofluids. Furthermore, in cancer patients, the ratio of CTCs to white blood cells is approximately 1:1 million. Recent developments in ctDNA detection have made it possible to identify ctDNA in the plasma of over 75% of end-stage cancer patients. In comparison, ctDNA was detected in less than 10% of glioma patients [49].

 ctDNA has been observed in the blood and CSF of adult patients with high-grade glioma. Research on ctDNA has primarily focused on identifying molecular deviations that hold diagnostic, prognostic, predictive, or monitoring significance. These ctDNA targets consist of IDH1 mutations, EGFRvIII mutation, loss of heterozygosity for 1p, 10q, 19q, and methylation of MGMT promoters, obtained from adult tissue biomarker literature. However, the current investigation of these ctDNA targets is limited [10]. Tumor hemorrhage, biopsy, and neuroanatomical location may contest the sensitivity of ctDNA as a diagnostic biomarker. For example, Wang et al. found that the proximity to CSF reservoirs was associated with increased detection of ctDNA in CSF [50]. Neurotrophic tyrosine receptor kinase (NTRK) fusion is commonly found in pediatric high-grade gliomas. The recent endorsement of the pan-tropomyosin receptor kinase inhibitor (TRK), larotrectinib, as a diagnostic tool for NTRK fusion products has augmented the attractiveness of non-invasive detection of NTRK fusions as a viable biomarker. The NTRK cfDNA has recently been employed as an examining and predictive biomarker for treatment response for agents targeting the mutational resistance mechanism [51].

ctDNA Detection Tools

Several methods have been utilized to evaluate ctDNA in blood or CSF, such as droplet digital PCR (ddPCR) amplification and magnetics (beads, emulsion, amplification, magnetics (BEAMing)), tagged-amplicon deep sequencing (TAm-Seq), cancer personalized profiling by deep sequencing (CAPP-Seq), whole genome bisulfite sequencing (WGBS-Seq), whole exome sequencing (WES), and whole genome sequencing (WGS) [49]. ddPCR has the ability to detect 0.01-1.0% of genomic materials, which is valuable in detecting potentially rare mutations and determining copy number variants. Nevertheless, it is restricted to assessing identified sequences [52].

BEAMing is an affordable and relatively sensitive screening technique for identifying common mutations. Combining PCR with flow cytometry, it is capable of detecting shifts at levels as low as 0.02% and shows outstanding matching with tissue [52]. CAPP-Seq is another method for detecting ctDNA alterations that involves analyzing huge genomic libraries and sequence signatures. The technique uses DNA oligonucleotides to statistically evaluate well-known tumor alterations and identify patient-specific changes. The method is equipped to detect numerous mutations in patients with the same cancer type and evaluate tumor heterogeneity. Previous studies demonstrated its ability to identify tumor burdens before medical imaging. It identifies a broad spectrum of mutational variants comprising significant insertions, deletions, single nucleotide variants, copy variants, and rearrangements, although fusions are not discernible through this method [46]. TAm-Seq enables an analysis that is both very specific and sensitive, capable of identifying DNA levels as low as 2% through the use of primers to tag and categorize genomic sequences. The method has a high sequencing flow, which can reduce sequencing time and expenses [53].

WES provides an expanded analysis and classification of all tumor mutations, which enables the identification of potential oncogenes and tumor suppressor genes. Nonetheless, its sensitivity may be relatively lower than other techniques because it only encompasses exome alterations. WGS assesses the complete tumor genome to recognize identified, harmful alterations, as well as unknown variant types. Despite exhibiting vast latent for a full evaluation of all tumor mutations, WGS is restricted by quality assurance, ethical reflections, time, and expenses. The interpretation of WGS findings can be intricate, frequently necessitating specialized centers [54]. WGBS-Seq is considered the gold standard in DNA methylation analysis due to its high accuracy and ability to provide a single cytosine measurement. Although it can detect partially methylated domains in cancer cells, the sensitivity of this technique can be affected by varying degrees of DNA degradation [55]. The levels, quantity, and quality of ctDNA can be altered by cell death induced by therapy. However, this phenomenon can be confounded by clonal hematopoiesis of indeterminate potential [49]. Compared to plasma and serum, the sensitivity of ctDNA detection is higher in CSF. One possible reason for this is that, even if partially disrupted, the blood-brain barrier restricts the passage of ctDNA from the primary brain tumor into the bloodstream [50].

cfRNA

cfRNA pertains to miRNAs found in biological fluids [56]. Lee et al. identified miRNA as intracellular non-coding RNA molecules consisting of 22 nucleotides. MiRNAs have a vital function in mediating post-transcriptional signal silencing in various cellular activities [57]. Exosomes, which are tiny membrane vesicles ranging from 30-100 nm, also possess miRNAs. In the context of cancer, the dysregulation of miRNAs can cause them to act as either tumor suppressors or oncogenes subject to their role. All high-grade gliomas (HGGs) contain miRNAs. In a study by Birks et al., the miRNA profiles of 24 pediatric CNS tumors were analyzed, revealing a distinct dysregulation of miR-129, miR-142-5p, and miR-25 [58].

The high turnover rate of tumor cells results in the upregulation of specific genes, causing the generation of large amounts of cfRNA. As a result, researchers have been able to identify corresponding alterations in the blood of cancer patients [57]. The limitation of miRNA analysis manifests in the lack of consistency when selecting reference genes. The quality and quantity of miRNAs obtained from sources such as plasma, serum, whole blood, and exosomes can vary during the separation process. Furthermore, some studies have limited sample sizes, which may result in untrustworthy outcomes. As a result, additional verification is necessary to guarantee the accuracy of miRNA isolation and quantification, as well as the methodology used for data analysis [59].

Exosomes

Exosomes, as a liquid biomarker, hold great promise as a prognostic or diagnostic methodology, carrying an array of miRNAs that exhibit substantial alterations between healthy controls and various cancers such as glioblastoma. The first discovery of exosomes occurred in sheep reticulocytes in 1983 and was named by Johnstone in 1987 [60]. It pertains to the vesicles that cells release and contains a large quantity of proteins, genetic information such as DNA and RNA, and other analytes [61]. Exosomes, which have a size ranging from 30 nm to 100 nm, are detectable in a variety of bodily fluids, such as plasma, saliva, urine, breast milk, hydrothorax, cerebrospinal fluid, semen, and others. Furthermore, they remain stable even in extreme pH conditions (pH = 1-13) and during freeze-thaw cycles. Since exosomes play a vital part in tumor growth and metastasis, additional research is necessary to comprehend their complex impact on cancer mechanisms [62].

The comparable nature of circulating exosomal miRNAs to tumor-derived miRNAs indicates that the former could be valuable for cancer screening. Furthermore, the inclusion of other genetic elements within exosomes can enhance research on tumor genetics. However, the method for obtaining exosomes is currently in the developmental stages, which is a major factor limiting research on exosomes [62]. On the other hand, cell-free messenger RNA (mRNA) is prone to rapid degradation in the bloodstream and originates from either living or dying tumor cells or CTCs. Whereas most of the cfDNA released comes from normal cells, implying ctDNA only represents a small portion of the cfDNA [14]. The cfDNA found in circulation is generally fragmented to lengths that range from 160 to 180 bp, which corresponds to DNA that is protected by nucleosomes and typically observed in apoptotic cells [63]. Exosomes that contain miRNAs have been detected in child brain tumors, which presents opportunities for prospective clinical trials that involve exosome analysis for monitoring disease advancement [64].

## Conclusions

Liquid biopsy has emerged as a promising non-invasive diagnostic tool in the field of neuro-oncology. Its potential to analyze biomarkers in body fluids, such as blood or CSF, which can provide valuable information about underlying cancer, including the diagnosis and prognosis of WHO grade 4 astrocytomas. With further research and development, liquid biopsy has the potential to revolutionize the way we diagnose and treat cancer, especially in cases of aggressive malignant brain tumors. 
